# A Hard Hit

**Published:** 1857-06

**Authors:** 


*A Hard Hit.*—It will be recollected that the late president of the American Medical Association had, a few years since, but a very small opinion of the uses of clinical instruction. He has recently changed, to some extent, his views on that subject, and announces clinical instruction at St. Mary's Hospital, Detroit, to which no student can be admitted unless he has attended one course of lectures at Ann Arbor. The idea of a clinical department located only forty miles from the school to which it is attached is novel, to say the least. Commenting thereon, the editor of the Medical Independent thus discourses:

"Here, then, is the Medical Department of the Michigan University, 'revised and improved' by the Board of Regents. To us it looks very much like 'the play of Hamlet,' with '*Hamlet*' left out, and before the last *rendering* is complete we venture to predict that it will be more appropriately named *the Regents folly*—in one act!

"But who are to represent the most practical department in this clinical teaching? Why are the 'Professors of Theory and Practice,' and the 'Professor of Surgery,' left out of the programme? Both are residents of the city, and both are zealous and competent teachers. Why are these departments represented by an *emeritus* professor, *assisted* by the Professor of Materia Medica? This is the way by which the Board of Regents propose to supply this '*important want* of the medical neophyte;' a professor in the institution is to be permitted to discharge his duties *by proxy.* May not the Professor of Surgery or Theory and Practice, with as much propriety associate with himself some *proxy*, from whose zeal and ability he can derive important aid in giving his winter course at Ann Arbor?

"If such are the only inducements offered by this 'appurtenance' to the University, is it reasonable to suppose that in addition to the six months of laborious study through the *winter course*, they will come here, into the city, during *the heat of summer*, to complete the curriculum?

"Can the Medical Department of the *Michigan University*, with such comparatively meagre clinical advantages, hope to compete successfully with schools wherein the *theoretical is associated and blended with the practical?* The great utility of clinical instruction consists chiefly in the fact, that it is *made to illustrate principles*, to familiarize the student with the physiognomy, or sensible aspects of disease, as well as its pathology, and the modus operandi of remedies.

"A few years since branches of the University were established in different parts of the State, the constant nursing of which crippled the University fund, and involved the institution in financial embarrassment. We have reason to fear that this last experiment—this *Branch of the Medical Department*—has been established upon the same short-sighted policy, the same want of practical knowledge and appreciation of the real object to be accomplished.

"There is one feature in the announcement of this summer arrangement which may surprise some of our readers, and though we may incur the risk of being considered invidious, its import gives additional strength to our views respecting this—at best—doubtful experiment of the Regents, and, therefore, merits a passing notice. It is very well known that the *emeritus* Professor who has received the appointment of 'clinical instructor,' has held *peculiar* views respecting *the utility* of hospital advantages; taking the ground that '*clinical observations* may be *most* successfully and profitably made *under the supervision and direction of* a *private preceptor*,' and that 'a *thorough* knowledge of the principles and elements of the science of medicine *should precede any attempt* at their practical application.'

"*Now* he speaks of these proffered advantages at St. Mary's Hospital, as an '*important want* of the medical neophyte.'

"What has so completely metamorphosed his published opinions on this subject?

"Why the Board of Regents should have chosen a man who has long entertained and published opinions entirely opposed to hospital instruction, is a question which time will answer. R."

---

We have received from Dr. John G. Orton, of Binghampton, an excellently devised book of record, for tabulating the statistics of medical practice. It is superior by far to any other, and offers a very convenient form for a history of the diseases seen in practice. It is designed by Dr. Orton, as one of the committee of the State Society on Medical Statistics, and we presume that an application to him would secure a copy at slight cost. Our impracticable friend and neighbor, Dr. Congar, made a similar attempt last year, but Dr. Orton's will be found much more ready, and better adapted to its purpose. It would be but little labor for the physician, using the "Physicians Diary," published by Messrs. Phinney & Co., of this city, to tabulate from it at the end of the month a very satisfactory record on one of the tables designed by Dr. Orton. If all our many physicians would do so, great good wonld be gained by the collection of a valuable mass of statistics.

---

*New York Journal of Medicine.*—A change in the editorial department of this journal is announced, The senior editor, Dr. S. S. Purple, has resigned his charge, and as Dr. Bulkley's connection was intended to be but temporary, Dr. Stephen Smith remains sole editor. Under his able administration the Journal will doubtless maintain the high rank it has hitherto held in medical periodical literature. We rejoice to learn that "circumstances of a favorable character, relating to his duties as a private practitioner," have required Dr. Purple to withdraw from the editorial chair.—*Boston Medical & Surgical Journal.*

*Sir Charles Locock.*—Her Majesty, Queen Victoria, has been graciously pleased to bestow the honors of Knighthood on her chief accoucheur, Dr. Locock. Mr. Punch suggests, however, that this distinguished gentleman deserved, after his arduous duties, to have been elevated to the peerage under the title of Lord Deliver us!

Some one down east, who dare not give his name, sends us the following Mrs. Partington. The old lady attempted to repeat the homœopathic precept, "Similia Similibus Curantur." She rendered it "Simia simiis curantur."

We may as well make our little couplet of jokes a triplet, by calling the attention of our readers to the fact that in the whole range of botany there is no flower which so much resembles *diabetes mellitus* as the *everlasting sweet pea.*

---

*Medical Miscellany.*—The Association of Medical Superintendents of Institutions for the Insane, which held its annual meeting in New York, last week, adjourned on Friday evening.

The sum of $50,000 has been appropriated by the Massachusetts Legislature, to finish the buildings of the third State Insane Asylum, at Northampton.

The number of graduates at the University of Pennsylvania, the present season, was 149.

A State Medical Society has been formed in Mississippi, and at its first meeting recently, Dr. W. Gadbury was chosen President.

Dr. C. V. Blaney has been appointed Visiting Physician to the U. S. Marine Hospital, at Chicago, and Dr. J. C. Nott to the Marine Hospital at Mobile.

Dr. Edward Banks, of Clinton, Miss., has presented to the Museum of the New Orleans School of Medicine, an interesting specimen, consisting of about 14 inches of the lower part of the small intestine of a child, so completely *stuffed* with large worms, as to entirely obstruct the canal. The gut is absolutely distended, and the blood-vessels are in the highest state of congestion.—*Boston Med. & Surg. Journal.*

